# Social Support Changes in Response to Retirement

**DOI:** 10.1093/geroni/igaa057.1429

**Published:** 2020-12-16

**Authors:** Brittany King

**Affiliations:** Florida State University, Tallahassee, Florida, United States

## Abstract

Retirement is an influential life course transition, that has potential to impact overall well-being as well as our social lives. The extent to which our social lives are changed is dependent upon the resources available both pre- and post-retirement. Research on retirement has focused on how well-being is changed, through measures such as depression, and conceptualize social support as a resource that can help offset some of the associated negative consequences. However, it is unclear how that resource of social support is itself being impacted. This study uses 2008-2016 Health and Retirement Study (HRS) data to assesses if how social support changes is dependent upon timing of retirement or whether the individual was forced to retire (N=1,933). Ordinary least squares regression (OLS) and marginal effects are used to assess the change in support and to test if the effects differentially impact certain groups. Preliminary results from this study show that men who have been retired for two waves report a significant negative change (p<0.05) in their in-person interactions with children, whereas women who have been retired for the same amount of time report a significant positive change (p<0.01) in their interactions with children. Additionally, women who are forced to retire report a significant (p<0.05) increase in their in-person interactions with children. These findings suggest that factors such as timing of retirement and forced retirement are important factors in understanding how received social support changes.

